# Supplementary material for: P-body proteins regulate transcriptional rewiring to promote DNA replication stress resistance
Source: Nat Commun. 2017 Sep 15;8:558. doi: 10.1038/s41467-017-00632-2 (PMC5601920; doi:10.1038/s41467-017-00632-2)
Supplement: Supplementary file 8 — Supplementary Information [file 41467_2017_632_MOESM8_ESM.pdf]

Description of Supplementary Files

File Name: Supplementary Information
Description: Supplementary Figures, Supplementary Tables and Supplementary References

File Name: Supplementary Data 1
Description: RNAseq data related to Figure 1

File Name: Supplementary Data 2
Description: GO term enrichment data related to Figure 1

File Name: Supplementary Data 3
Description: HU sensitivity suppressor screen data related to Figure 2

File Name: Supplementary Data 4
Description: RNAseq data related to Figure 6

File Name: Supplementary Data 5
Description: Fitness data related to Figure 6.

File Name: Supplementary Software 1
Description: R scripts and Cellprofiler pipelines used for the analysis of the data generated in this manuscript

File Name: Peer Review File

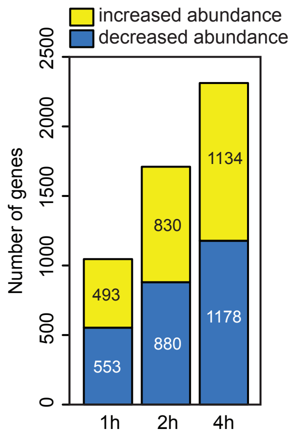

Supplementary Fig. 1. The number of genes induced or repressed upon HU exposure in WT cells. The number of differentially expressed genes was determined using the Tuxedo protocol.

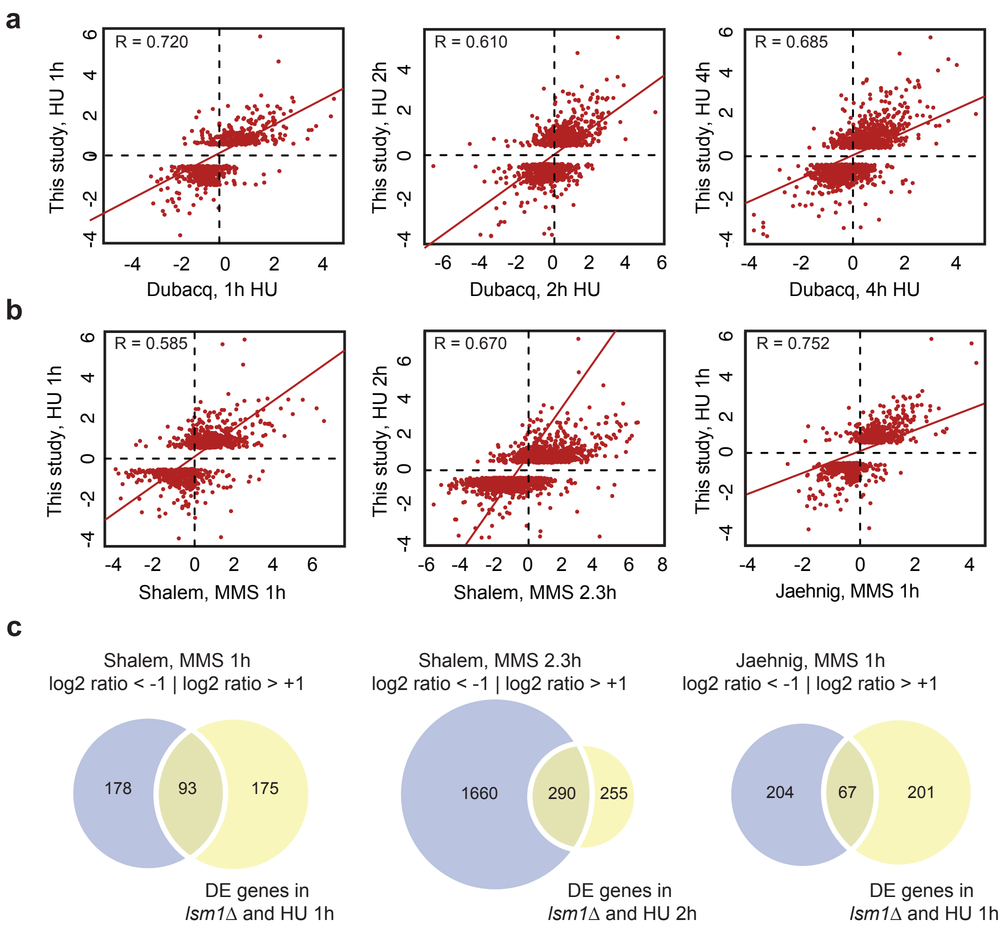

Supplementary Fig. 2. Lsm1 regulates RNA abudance of HU-responsive genes as well as more general DNA replication stress genes. (a) Correlation between the expression ratios for the differentially expressed genes identified in WT cells in this study upon HU exposure and the study of Dubacq et al. (2006) at 1, 2 and 4 hours in HU. **(b)** Correlation between the expression ratios for the differentially expressed genes identified in WT cells in this study upon HU exposure and WT cells in MMS in the study of Shalem et al. (2008) or Jaehnig et al. (2013). **(c)** Venn Diagram representing the overlap between the genes showing log2 expression ratios inferior to -1 or superior to +1 in the study of Shalem et al. (2008) or Jaehnig et al. (2013) and the genes showing differential expression upon HU exposure and in *lsm1*Δ cells in this study. DE: Differentially Expressed

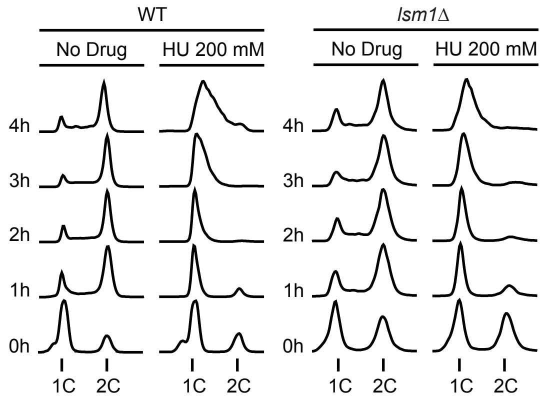

Supplementary Fig. 3. Cell-cycle progression of the WT and *lsm1*Δ strain under normal growth or in presence of HU. Flow cytometry histograms of DNA contents in wild type and lsm1Δ cells. Cells were arrested in G1 and then released into S phase in the absence or presence of HU. The positions of cells with 1C and 2C DNA contents are indicated.

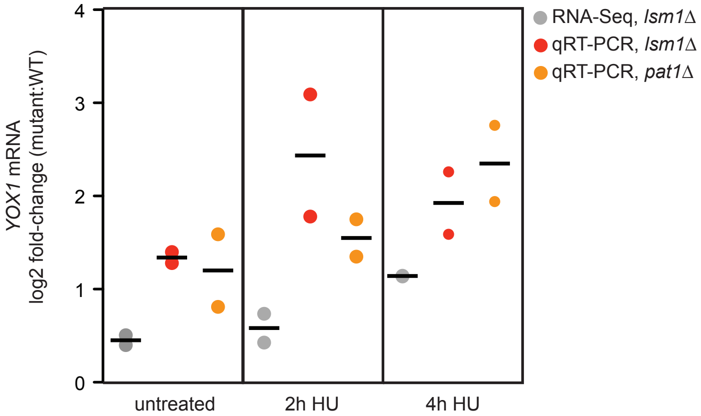

Supplementary Fig. 4. *YOX1* RNA abudance increases in *lsm1*Δ and *pat1*Δ cells. log2 expression ratios comparing *YOX1* mRNA levels in WT and *lsm1*Δ cells obtained by RNA-Seq (grey) or qRT-PCR (red) or log2 expression ratios comparing *YOX1* mRNA levels in WT and *pat1*Δ cells obtained by qRT-PCR (orange). The means and two biological replicates are shown. Each qRT-PCR biological replicate included at least 2 technical replicates.

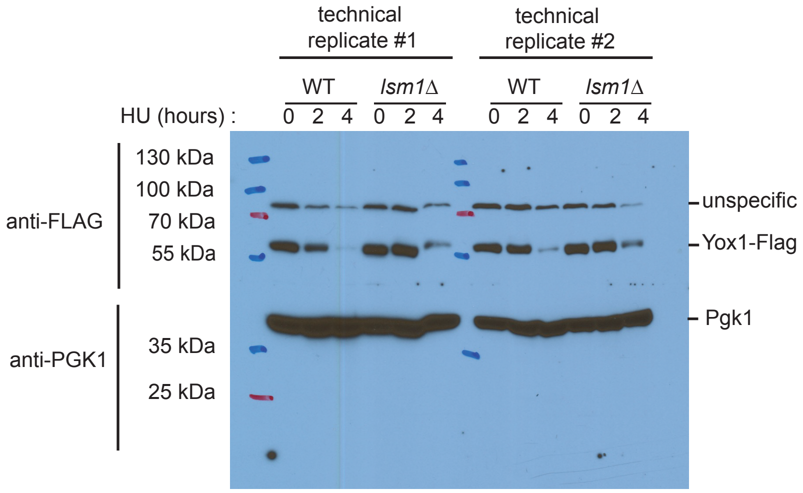

Supplementary Fig. 5. Full immuno-blot image related to Fig. 5b. Yox1-FLAG protein abundance was probed using anti-FLAG antibody. Pgk1 was used as loading control.

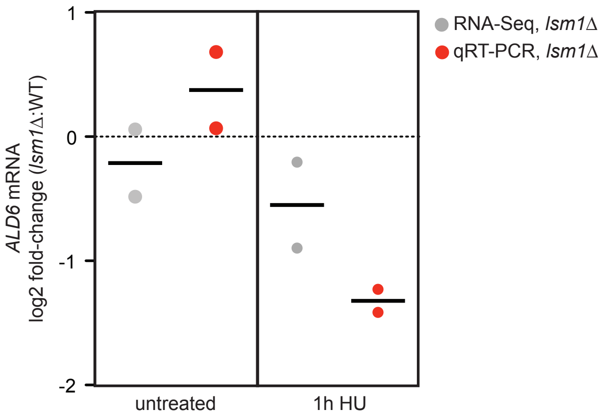

Supplementary Fig. 6. *ALD6* mRNA abundance decreases in *lsm1*Δ cells. log2 expression ratios comparing *ALD6* mRNA levels in WT and *lsm1*Δ cells obtained by RNA-Seq (grey) or qRT-PCR (red). The means and two biological replicates are shown. Each qRT-PCR biological replicate included at least 2 technical replicates.

Supplementary Table 1. Comparison of RNA-seq results obtained with Cuffdiff, EBSeq or edgeR

		down-regulated genes		up-regulated genes	
method	condition	number of DE genes identified	overlap with cuffdiff (%)	number of DE genes identified	overlap with cuffdiff (%)
cuffdiff	untreated	258	NA	333	NA
EBSeq		564	198 (35%)	521	208 (40%)
edgeR		703	236 (34%)	800	305 (38%)
cuffdiff	1h HU	332	NA	499	NA
EBSeq		682	265 (39%)	867	382 (44%)
edgeR		762	319 (42%)	911	455 (50%)
cuffdiff	2h HU	686	NA	911	NA
EBSeq		817	514 (63%)	959	697 (73%)
edgeR		1509	666 (44%)	1578	888 (57%)
cuffdiff	4h HU	1051	NA	1203	NA
EBSeq		1026	793 (77%)	1085	860 (79%)
edgeR		1753	1024 (58%)	1887	1176 (62%)

DE : Differentially Expressed

Supplementary Table 2. Expression data from cuffdiff, EBSeq and edgeR for the genes identified in Fig. 2

		cuffdiff			EBSeq		edgeR		
condition	gene	log2 FC	p-value	FDR corrected p-value	log2 FC	posterior probability [1]	log2 FC	p-value	FDR corrected p-value
untreated	*ACF4*	0,185	3,70E-01	6,76E-01	0,186	1,18E-12	0,190	2,09E-01	3,99E-01
	ARL3	0,497	1,62E-02	1,12E-01	0,497	1,38E-08	0,499	8,44E-04	6,61E-03
	HHT1	1,010	5,00E-05	1,63E-03	1,011	1,00E+00	1,014	2,30E-14	3,00E-12
	RRS1	0,682	1,50E-04	3,99E-03	0,683	1,00E+00	0,686	1,26E-08	5,08E-07
	TMA16	0,941	5,00E-05	1,63E-03	0,942	1,00E+00	0,946	1,65E-14	2,19E-12
	YOX1	0,454	1,88E-02	1,22E-01	0,454	1,21E-07	0,458	9,92E-04	7,47E-03
1h HU	*ACF4*	0,336	1,33E-01	3,57E-01	0,336	2,68E-12	0,325	4,93E-02	1,39E-01
	ARL3	0,437	4,29E-02	1,76E-01	0,437	1,05E-09	0,426	1,04E-02	4,34E-02
	HHT1	1,801	5,00E-05	1,11E-03	1,802	1,00E+00	1,789	5,49E-23	1,01E-20
	RRS1	0,386	7,17E-02	2,46E-01	0,387	1,78E-01	0,377	9,04E-02	2,17E-01
	TMA16	1,079	5,00E-05	1,11E-03	1,079	1,00E+00	1,068	1,86E-09	7,75E-08
	YOX1	0,958	5,00E-05	1,11E-03	0,956	1,00E+00	0,947	7,49E-07	1,53E-05
2h HU	*ACF4*	0,510	5,60E-03	2,74E-02	0,500	7,25E-07	0,500	2,94E-04	1,06E-03
	ARL3	0,645	4,50E-04	3,60E-03	0,635	3,07E-01	0,631	1,37E-08	1,12E-07
	HHT1	1,321	5,00E-05	5,58E-04	1,313	1,00E+00	1,309	7,11E-40	1,24E-37
	RRS1	0,647	1,50E-04	1,47E-03	0,638	1,00E+00	0,634	7,96E-12	1,07E-10
	TMA16	1,034	5,00E-05	5,58E-04	1,024	1,00E+00	1,021	5,04E-25	3,22E-23
	YOX1	0,572	1,25E-03	8,27E-03	0,563	4,38E-04	0,560	1,26E-05	6,01E-05
4h HU	*ACF4*	0,630	1,45E-03	6,30E-03	0,623	1,44E-04	0,631	2,10E-06	8,16E-06
	ARL3	0,425	1,25E-02	3,77E-02	0,420	3,81E-07	0,427	2,66E-04	7,54E-04
	HHT1	1,356	5,00E-05	3,47E-04	1,351	1,00E+00	1,355	3,19E-55	4,82E-53
	RRS1	0,746	5,00E-05	3,47E-04	0,741	1,00E+00	0,745	9,25E-17	1,19E-15
	TMA16	1,233	5,00E-05	3,47E-04	1,226	1,00E+00	1,231	4,60E-32	1,89E-30
	YOX1	1,141	5,00E-05	3,47E-04	1,134	1,00E+00	1,139	1,64E-23	4,00E-22

FC : Fold-Change; Red shading indicates statistically supported differential expression

[1] Values superior or equal to 0.95 are considered significant for posterior probability in EBSeq

Supplementary Table 3. YETFASCO scanning results for *ALD6* and *ICS2* promoter regions

Gene promoter scanned	Transcription factor	Coordinates (relative to START codon)		Strand	% of Max score	Motif	YETFASCO motif ID
ALD6	Yox1	-987	-978	-	76,8	GTTGATTGGG	#2196
		-855	-846	-	75,4	GTTAATGGTG	#2196
		-660	-651	+	77	CTAAATGACT	#2196
		-298	-289	-	81,6	AGTAATTGGC	#2196
		-296	-289	+	100	CAATTACT	#453
ICS2	Yox1	-162	-155	-	97,3	TAATTACA	#453
		-545	-536	-	76,7	GTTAATGACT	#2196
		-303	-296	-	76,3	TAATTAGG	#453
		-302	-295	-	80,6	ATAATTAG	#498
		-268	-259	-	78,3	GTTTATTACG	#2196
ALD6	Mcm1	-823	-816	-	84,2	TTCGTCAA	#1369
		-384	-375	+	80,2	CCCCAAACGG	#2139
		-383	-374	+	93,8	CCCAAACGGG	#2139
		-344	-329	-	79,9	CTTCCCAAAGAGGAAA	#1015
		-344	-329	-	78,2	CTTCCCAAAGAGGAAA	#1485
		-344	-330	-	86	TTCCCAAAGAGGAAA	#1298
		-344	-329	-	78,7	CTTCCCAAAGAGGAAA	#1544
		-341	-330	-	86,2	TTCCCAAAGAGG	#1543
		-341	-332	-	85,1	CCCAAAGAGG	#2139
		-337	-330	-	78,6	TTCCCAAA	#1369
ICS2	Mcm1	-993	-986	+	80,5	TCCGCAAT	#1369
		-924	-917	-	91,1	TTCGCAAT	#1369
		-580	-573	+	82,9	AAAATAGG	#1368
		-304	-297	-	82,9	AATTAGGT	#1367
		-303	-296	-	85,4	TAATTAGG	#1368
		-40	-33	+	79,6	TTCGTTAT	#1369
		-303	-296	-	85,4	TAATTAGG	#1368
		-40	-33	+	79,6	TTCGTTAT	#1369

Supplementary Table 4. Strains used in this study.

Lab ID	Strain name	Genotype	Source
BY4741	WT	*MAT**a** leu2Δ0 his3Δ1 ura3Δ0 met15Δ0*	1
RLKY6	lsm1Δ	*MATα lsm1Δ::kanMX can1Δ::STE2pr-his5 lyp1Δ leu2Δ0 his3Δ1 ura3Δ0*	C. Boone
JTY16	lsm1Δ	*MAT**a** leu2Δ0 his3Δ1 ura3Δ0 met15Δ0*	2
RLKY15	pat1Δ	*MATα pat1Δ::natMX can1Δ::STE2pr-his5 lyp1Δ::STE3pr-LEU2*	C. Boone
RLKY24	WT + pBY011	*MAT**a** pBY011[GAL1-10pr-] leu2Δ0 his3Δ1 ura3Δ0 met15Δ0*	this study
RLKY27	WT + pBY011-YOX1	*MAT**a** pBY011[GAL1-10pr-YOX1] leu2Δ0 his3Δ1 ura3Δ0 met15Δ0*	this study
RLKY53	lsm1Δ + pBY011	*MAT**a** lsm1Δ::kanMX pBY011[GAL1-10p-] leu2Δ0 his3Δ1 ura3Δ0 met15Δ0*	this study
RLKY54	lsm1Δ + pBY011-YOX1	*MAT**a** lsm1Δ::kanMX pBY011[GAL1-10pr-YOX1] leu2Δ0 his3Δ1 ura3Δ0 met15Δ0*	this study
RLKY55	pat1Δ + pBY011	*MAT**a** pat1Δ::kanMX pBY011[GAL1-10pr-] leu2Δ0 his3Δ1 ura3Δ0 met15Δ0*	this study
RLKY56	pat1Δ + pBY011-YOX1	*MAT**a** pat1Δ::kanMX pBY011[GAL1-10p-YOX1] leu2Δ0 his3Δ1 ura3Δ0 met15Δ0*	this study
RLKY57	yox1Δ	*MAT**a** yox1Δ::kanMX leu2Δ0 his3Δ1 ura3Δ0 met15Δ0*	3
RLKY58	yhp1Δ	*MAT**a** yhp1Δ::kanMX leu2Δ0 his3Δ1 ura3Δ0 met15Δ0*	3
RLKY60	lsm1Δ yox1Δ	*MAT**a** lsm1Δ::natMX yox1Δ::kanMX can1Δ::STE2pr-his5 lyp1Δ leu2Δ0 his3Δ1 ura3Δ0 lys2Δ0*	this study
RLKY61	lsm1Δ yhp1Δ	*MAT**a** lsm1Δ::natMX yhp1Δ::kanMX can1Δ::STE2pr-his5 lyp1Δ leu2Δ0 his3Δ1 ura3Δ0 lys2Δ0*	this study
RLKY63	pat1Δ yox1Δ	*MAT**a** pat1Δ::natMX yox1Δ::kanMX can1Δ::STE2pr-his5 lyp1Δ::STE3pr-LEU2 leu2Δ0 his3Δ1 ura3Δ0 lys2Δ0*	this study
RLKY64	pat1Δ yhp1Δ	*MAT**a** pat1Δ::natMX yhp1Δ::kanMX can1Δ::STE2pr-his5 lyp1Δ::STE3pr-LEU2 leu2Δ0 his3Δ1 ura3Δ0 lys2Δ0*	this study
RLKY66	xrn1Δ	*MATα xrn1Δ::natMX can1Δ::STE2pr-his5 lyp1Δ::STE3pr-LEU2 leu2Δ0 his3Δ1 ura3Δ0*	C. Boone
RLKY81	xrn1Δ yox1Δ	*MATα xrn1Δ::natMX yox1Δ::kanMX can1Δ::STE2pr-his5 lyp1Δ::STE3pr-LEU2 leu2Δ0 his3Δ1 ura3Δ0 lys2Δ0*	this study
RLKY82	xrn1Δ yhp1Δ	*MATα xrn1Δ::natMX yhp1Δ::kanMX can1Δ::STE2pr-his5 lyp1Δ::STE3pr-LEU2 leu2Δ0 his3Δ1 ura3Δ0 lys2Δ0*	this study
RLK43	YOX1-MS2 Lsm1-mCherry	*MAT**a** /MATα YOX1-MS2/YOX1 LSM1/LSM1-mCherry-natMX pMS2-CP-GFP(x3) leu2Δ0/leu2Δ0 his3Δ1/his3Δ1 ura3Δ0/ura3Δ0 lys2Δ0/LYS2 met15Δ0/MET15*	this study
RLK45	YOX1-MS2 Pat1-mCherry	*MAT**a** /MATα YOX1-MS2/YOX1 PAT1/PAT1-mCherry-natMX pMS2-CP-GFP(x3 leu2Δ0/leu2Δ0 his3Δ1/his3Δ1 ura3Δ0/ura3Δ0 lys2Δ0/LYS2 met15Δ0/MET15)*	this study
RLKY72	xrn1Δ YOX1-MS2 Lsm1-mCherry	*MAT**a** /MATα xrn1Δ::kanMX/xrn1Δ::kanMX YOX1-MS2/YOX1 LSM1/LSM1-mCherry-natMX pMS2-CP-GFP(x3) leu2Δ0/leu2Δ0 his3Δ1/his3Δ1 ura3Δ0/ura3Δ0 lys2Δ0/LYS2 met15Δ0/MET15*	this study
RLKY71	xrn1Δ YOX1-MS2 Pat1-mCherry	*MAT**a** /MATα xrn1Δ::kanMX/xrn1Δ::kanMX YOX1-MS2/YOX1 PAT1/PAT1-mCherry-natMX pMS2-CP-GFP(x3) leu2Δ0/leu2Δ0 his3Δ1/his3Δ1 ura3Δ0/ura3Δ0 lys2Δ0/LYS2 met15Δ0/MET15*	this study
RLKY74	YOX1 Lsm1-mCherry	*MAT**a** /MATα YOX1/YOX1 LSM1/LSM1-mCherry-natMX pMS2-CP-GFP(x3) leu2Δ0/leu2Δ0 his3Δ1/his3Δ1 ura3Δ0/ura3Δ0 lys2Δ0/LYS2 met15Δ0/MET15*	this study

RLKY75	YOX1 Pat1-mCherry	*MAT**a** /MATα YOX1/YOX1 PAT1/PAT1-mCherry-natMX pMS2-CP-GFP(x3) leu2Δ0/leu2Δ0 his3Δ1/his3Δ1 ura3Δ0/ura3Δ0 lys2Δ0/LYS2 met15Δ0/MET15*	this study
RLKY76	xrn1Δ YOX1 Lsm1-mCherry	*MAT**a** /MATα xrn1Δ::kanMX/xrn1Δ::kanMX YOX1/YOX1 LSM1/LSM1-mCherry-natMX pMS2-CP-GFP(x3) leu2Δ0/leu2Δ0 his3Δ1/his3Δ1 ura3Δ0/ura3Δ0 lys2Δ0/LYS2 met15Δ0/MET15*	this study
RLKY77	xrn1Δ YOX1 Pat1-mCherry	*MAT**a**/MATα xrn1Δ::kanMX/xrn1Δ::kanMX YOX1/YOX1 PAT1/PAT1-mCherry-natMX pMS2-CP-GFP(x3) leu2Δ0/leu2Δ0 his3Δ1/his3Δ1 ura3Δ0/ura3Δ0 lys2Δ0/LYS2 met15Δ0/MET15*	this study
RLKY80	HTA2-mCherry	*MATα HTA2-mCherry-hphMX can1Δ::STE2pr-LEU2 lyp1Δ leu2Δ0 his3Δ1 ura3Δ0*	this study
RLKY86	YOX1-GFP HTA2-mCherry	*MAT**a** HTA2-mCherry-hphMX YOX1-GFP- GFP-HIS3MX can1Δ::STE2pr-LEU2 lyp1Δ leu2Δ0 his3Δ1 ura3Δ0*	this study
RLKY87	lsm1Δ YOX1-GFP HTA2-mCherry	*MAT**a** lsm1Δ::kanMX HTA2-mCherry-hphMX YOX1-GFP- GFP-HIS3MX can1Δ::STE2pr-LEU2 lyp1Δ leu2Δ0 his3Δ1 ura3Δ0*	this study
NTY13	RPL39pr-tdTomato	*MATα can1Δ::RPL39pr-tdTomato-CaURA3::STE2pr-LEU2 lyp1Δ leu2Δ0 his3Δ1 ura3Δ0*	4
RLKY83	YOX1-GFP RPL39pr-tdTomato	*MAT**a** YOX1-GFP-HIS3MX can1Δ::RPL39pr-tdTomato-CaURA3::STE2pr-LEU2 lyp1Δ leu2Δ0 his3Δ1 ura3Δ0*	this study
RLKY84	lsm1Δ YOX1-GFP RPL39pr-tdTomato	*MAT**a** lsm1Δ::kanMX YOX1-GFP-HIS3MX can1Δ::RPL39pr-tdTomato-CaURA3::STE2pr-LEU2 lyp1Δ leu2Δ0 his3Δ1 ura3Δ0*	this study
RLKY104	pry3Δ (query)	*MATα pry3Δ::natMX can1Δ::STE2pr-his5 lyp1Δ leu2Δ0 his3Δ1 ura3Δ0 met15Δ0*	C. Boone
RLKY105	ald6Δ (query)	*MATα ald6Δ::natMX can1Δ::STE2pr-his5 lyp1Δ leu2Δ0 his3Δ1 ura3Δ0 met15Δ0*	C. Boone
RLKY106	rad54Δ (query)	*MATα rad54Δ::natMX can1Δ::STE2pr-his5 lyp1Δ leu2Δ0 his3Δ1 ura3Δ0 met15Δ0*	C. Boone
RLKY107	yml131WΔ (query)	*MATα yml131wΔ::natMX can1Δ::STE2pr-his5 lyp1Δ leu2Δ0 his3Δ1 ura3Δ0 met15Δ0*	C. Boone
RLKY108	mmr1Δ (query)	*MATα mmr1Δ::natMX can1Δ::STE2pr-his5 lyp1Δ leu2Δ0 his3Δ1 ura3Δ0 met15Δ0*	C. Boone
RLK109	ura3Δ (query)	*MATα ura3Δ::natMX can1Δ::STE2pr-his5 lyp1Δ leu2Δ0 his3Δ1 ura3Δ0 met15Δ0*	C. Boone
RLKY110	ald6Δ	*MAT**a** ald6Δ::kanMX leu2Δ0 his3Δ1 ura3Δ0 met15Δ0*	3
RLKY111	YOX1-FLAG	*MAT**a** YOX1-FLAG-KanMX leu2Δ0 his3Δ1 ura3Δ0 met15Δ0*	this study
RLKY112	lsm1Δ YOX1-FLAG	*MAT**a** lsm1Δ::URA3MX YOX1-FLAG-KanMX leu2Δ0 his3Δ1 ura3Δ0 met15Δ0*	this study

	**All strains in Figure 2 have the following genotypes:**<br>*MAT**a** lsm1Δ::natMX xxxΔ::kanMX can1Δ::STE2pr-his5 lyp1Δ leu2Δ0 his3Δ1 ura3Δ0 lys2Δ0*<br>*MAT**a** pat1Δ::natMX xxxΔ::kanMX can1Δ::STE2pr-his5 lyp1Δ::STE3pr-LEU2 leu2Δ0 his3Δ1 ura3Δ0 lys2Δ0*	this study

	**All strains in Figure 6 have the following genotypes:**<br>*MAT***a** *xxxΔ::kanMX yyyΔ::natMX leu2Δ0 his3Δ1 ura3Δ0 lyp1Δ0 can1Δ0::STE2pr-SpHIS5*	this study
	**All strains in Figure 7a-b have the following genotypes:**<br>*MAT***a** *lsm1Δ::kanMX leu2Δ0 his3Δ1 ura3Δ0 met15Δ0 pBY011[GAL1-10pr-]*<br>*MAT***a** *lsm1Δ::kanMX leu2Δ0 his3Δ1 ura3Δ0 met15Δ0 pBY011[GAL1-10pr-XXX]*	this study
	**All strains in Figure 7f-g have the following genotypes:**<br>*MAT***a** *xxxΔ::NatMX RNR3-GFP-HIS3MX can1pr::RPL39pr-tdTomato-CaURA3 can1Δ::STE2pr-LEU2 leu2Δ0 his3Δ1 ura3Δ0 met15Δ0 lyp1Δ0*	this study

References

1. Brachmann, C. B. *et al.* Designer deletion strains derived from Saccharomyces cerevisiae S288C: a useful set of strains and plasmids for PCR-mediated gene disruption and other applications. *Yeast* **14,** 115–32 (1998).

2. Tkach, J. M. *et al.* Dissecting DNA damage response pathways by analysing protein localization and abundance changes during DNA replication stress. *Nat. Cell Biol.* **14,** 966–76 (2012).

3. Giaever, G. *et al.* Functional profiling of the Saccharomyces cerevisiae genome. *Nature* **418,** 387–391 (2002).

4. Hendry, J. A., Tan, G., Ou, J., Boone, C. & Brown, G. W. Leveraging DNA damage response signaling to identify yeast genes controlling genome stability. *G3 (Bethesda).* **5,** 997–1006 (2015).
